# Unraveling the transcriptional regulation of *TWIST1* in limb development

**DOI:** 10.1371/journal.pgen.1007738

**Published:** 2018-10-29

**Authors:** Naama Hirsch, Reut Eshel, Reut Bar Yaacov, Tal Shahar, Fania Shmulevich, Idit Dahan, Noam Levaot, Tommy Kaplan, Darío G. Lupiáñez, Ramon Y. Birnbaum

**Affiliations:** 1 Department of Life Sciences, Ben-Gurion University of the Negev, Beer-Sheva, Israel; 2 Center for Evolutionary Genomics and Medicine, Ben-Gurion University of the Negev, Beer-Sheva, Israel; 3 Department of Physiology and Cell Biology, Faculty of Health Sciences, Ben-Gurion University of the Negev, Beer-Sheva, Israel; 4 School of Computer Science and Engineering, The Hebrew University of Jerusalem, Jerusalem, Israel; 5 Max Planck Institute for Molecular Genetics, Berlin, Germany; 6 Institute for Medical and Human Genetics, Charité-Universitätsmedizin Berlin, Berlin, Germany; 7 Berlin-Brandenburg Center for Regenerative Therapies, Charité-Universitätsmedizin Berlin, Berlin, Germany; University of Chicago, UNITED STATES

## Abstract

The transcription factor *TWIST1* plays a vital role in mesoderm development, particularly in limb and craniofacial formation. Accordingly, haploinsufficiency of *TWIST1* can cause limb and craniofacial malformations as part of Saethre-Chotzen syndrome. However, the molecular basis of *TWIST1* transcriptional regulation during development has yet to be elucidated. Here, we characterized active enhancers in the *TWIST1-HDAC9* locus that drive transcription in the developing limb and branchial arches. Using available p300 and H3K27ac ChIP-seq data, we identified 12 enhancer candidates, located both within and outside the coding sequences of the neighboring gene, *Histone deacetyase 9 (HDAC9*). Using zebrafish and mouse enhancer assays, we showed that eight of these candidates have limb/fin and branchial arch enhancer activity that resemble *Twist1* expression. Using 4C-seq, we showed that the *Twist1* promoter region interacts with three enhancers (*eTw-5*, *6*, *7*) in the limb bud and branchial arch of mouse embryos at day 11.5. Furthermore, we found that two transcription factors, LMX1B and TFAP2, bind these enhancers and modulate their enhancer activity. Finally, using CRISPR/Cas9 genome editing, we showed that homozygous deletion of *eTw5-7* enhancers reduced *Twist1* expression in the limb bud and caused pre-axial polydactyly, a phenotype observed in Twist1^+/-^ mice. Taken together, our findings reveal that each enhancer has a discrete activity pattern, and together comprise a spatiotemporal regulatory network of *Twist1* transcription in the developing limbs/fins and branchial arches. Our study suggests that mutations in *TWIST1* enhancers could lead to reduced *TWIST1* expression, resulting in phenotypic outcome as seen with *TWIST1* coding mutations.

## Author summary

*TWIST1* haploinsufficiency can cause limb and craniofacial malformations, such as Saethre-Chotzen syndrome. However, the DNA regulatory elements that control *TWIST1* expression and prevent developmental abnormalities are not known. Here, we identified and characterized several *TWIST1* transcriptional enhancers. Each enhancer activates expression in a discrete sub-tissue of a limb or branchial arch and together correspond to a cluster of elements that mimic *TWIST1* spatiotemporal expression. Furthermore, two important transcription factors, LMX1B and TFAP2, were found to bind these enhancers and participate in their activity. Finally, homozygous deletion of three *Twist1* enhancers (*eTw5-7*) reduced *Twist1* expression in the limb bud and led to polydactyly, as seen in heterozygous *Twist1*-/+ mice. This study elucidates *TWIST1* regulatory elements and essential components of its transcriptional machinery, suggesting that alteration of these enhancers could lead to loss of function that may result in similar phenotypic outcomes as seen with *TWIST1* coding mutations.

## Introduction

Transcription regulation is central to coordinating limb bud patterning and craniofacial formation during embryonic development. On the molecular level, this transcriptional regulation includes gene regulatory elements, such as promoters and enhancers, the epigenetic state and spatial chromatin organization [1–3]. Enhancers are DNA sequences that are bound by transcription factors (TFs) in a sequence-specific manner. Enhancers are prevalent throughout the mammalian genome, and are found not only in intronic and intergenic regions but also in exonic sequences [4]. Mutations in enhancer sequences can explain the molecular basis of developmental disorders [5]. Indeed, large-scale human genetic studies have demonstrated that nucleotide variants within gene regulatory elements contribute to a wide spectrum of human traits and disorders, including limb and craniofacial disorders [6–8]. As an example, gain-of-function mutations in the Sonic hedgehog (*Shh)* limb enhancer lead to ectopic SHH expression in the anterior limb bud that causes polydactyly (extra finger) [9, 10]. Still, tissue-specific enhancers and their targeted genes during limb and craniofacial development have yet to be identified.

Twist family bHLH transcription factor 1 (*TWIST1)* encodes a TF whose activity is critical for mesodermal development, in particular during limb and craniofacial formation. *TWIST1* can act both as a repressor and an activator, depending on its dimerization state [11]. *TWIST1* regulates the expression of various TFs and signaling pathways in the developing limb bud and branchial arches, and serves as a direct regulator of the SHH pathway [12]. In the anterior limb bud, TWIST1 activates the transcription of *GLI Family Zinc Finger 3* (*GLI3*), which in turn inhibits the ectopic expression of *Heart and neural crest derivatives expressed 2* (*HAND2*) and activates *Aristaless-like homeobox 4* (*ALX4*), together forming a mutual antagonism loop which is central in SHH pathway initiation to ensure normal digit formation [13]. In mammalian development, *TWIST1* haploinsufficiency is associated with a range of limb and craniofacial malformations, including preaxial-polydactyly (PPD) [14, 15]. In humans, *TWIST1* mutations are associated with Saethre-Chotzen syndrome, an autosomal dominant craniosynostosis disorder that also exhibits distal limb malformation, such as PPD [16–18]. In *Twist1* homozygous mouse null mutant embryos, the growth of both forelimb and hindlimb buds is arrested early in development, although the hindlimb bud is less affected [19]. Ablation of *Twist1* activity in only the anterior mesenchyme of the forelimb bud leads to posteriorization of anterior skeletal elements, including mirror-image digit duplications and the acquisition of an ulnar-like morphology by the radius [20]. This phenotype is associated with down-regulation of *Alx*3/4 and *Gli3* in the anterior tissue of the forelimb bud, and parallel expansion of *Hoxd13* and *Gremlin 1* expression domains into the anterior region of the limb bud [20]. Thus, meeting a Twist1 dosage threshold is essential for normal limb and craniofacial development, indicating an important role for the transcriptional regulation of *TWIST1*.

In this study, we report the identification of enhancers that regulate *TWIST1* in distinct regions of the limb bud and branchial arch during embryonic development. We also describe two TFs, LMX1B and TFAP2, that participate in *TWIST1* enhancer activity, and show the *in vivo* effect of *Twist1* enhancers, whose deletion from the genome causes to reduction of *Twist1* expression and PPD, as seen in Twist1-/+ mice.

## Results

### Defining the *TWIST1* regulatory region and selection of enhancer candidates

**Fig 1:**
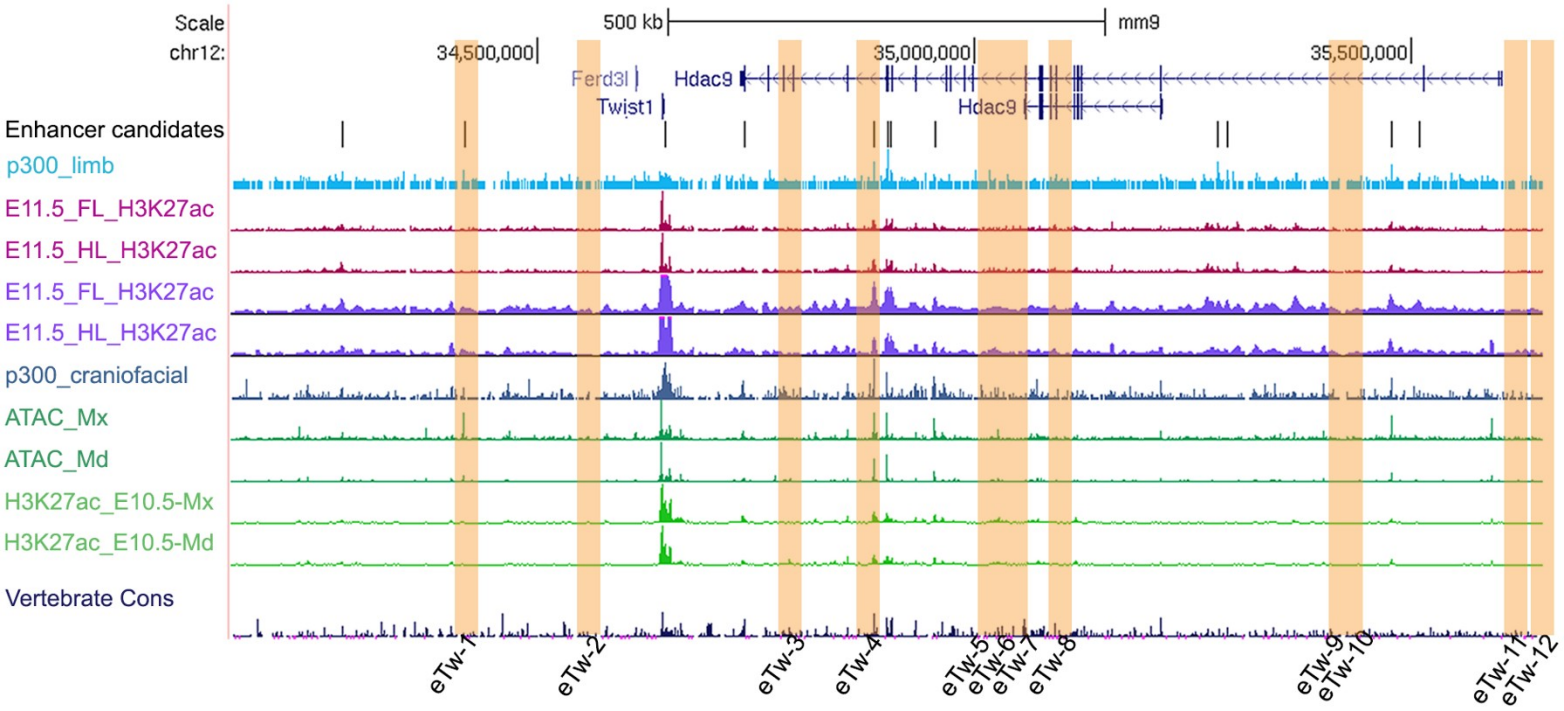
Identification of enhancer candidates in the *Twist1-Hdac9* locus. UCSC genome browser tracks of enhancer-associated marks (H3K27ac, p300) on mouse E11.5 whole limb, forelimb, hindlimb (blue for Visel et al 2009; red for Coteny et al. 2012 and purple for Andrey et al, 2017) and mouse E10.5/1E11.5 craniofacial tissues (green for Attanasio et al 2013; ATAC-seq and H3K27ac for Minoux et al., 2018). Selected enhancer candidates are marked by an orange rectangle [24–28].

Since *TWIST1* expression pattern is well conserved from zebrafish to humans, and as *TWIST1* and *HDAC9* share a synteny block [4], it is likely that *TWIST1* regulatory elements are also conserved and located in this region (hg19: chr7:18,050,988–19,741,484). Using PSYCHIC [21], a computational approach for analyzing Hi-C data and identifying promoter-enhancer interactions, we analyzed chromatin interactions from Hi-C data collected from various cell and tissue types [22, 23]. We found that *TWIST1* and *HDAC9* are located in the same topologically associate domain (TAD), which is conserved between various cell types and tissues. Since *HDAC9* is not expressed in the developing tissues where *TWIST1* is expressed, we proposed that *TWIST1* regulatory elements are likely located in this locus (S1 Fig). To identify *TWIST1* enhancers, we analyzed ATAC-seq and enhancer-associated ChIP-seq datasets (p300, H3K27ac) of mouse limb buds and branchial arches [24–28]. We focused our analysis on the *TWIST1-HDAC9* locus and selected candidate sequences that are marked by both p300 and H3K27ac (Fig 1). The p300 ChIP-peaks and the degree of evolutionary conservation were used to determine the lengths of the putative enhancer sequences (Fig 1). Accordingly, we identified 12 enhancer candidates, corresponding to seven intergenic, two intronic and three exonic sequences (based on *HDAC9* NM_058176), that might regulate *TWIST1* transcription during limb and brachial arch development (Fig 1, S1 Table).

### Identifying active enhancers *in vivo* using a zebrafish enhancer assay

**Fig 2:**
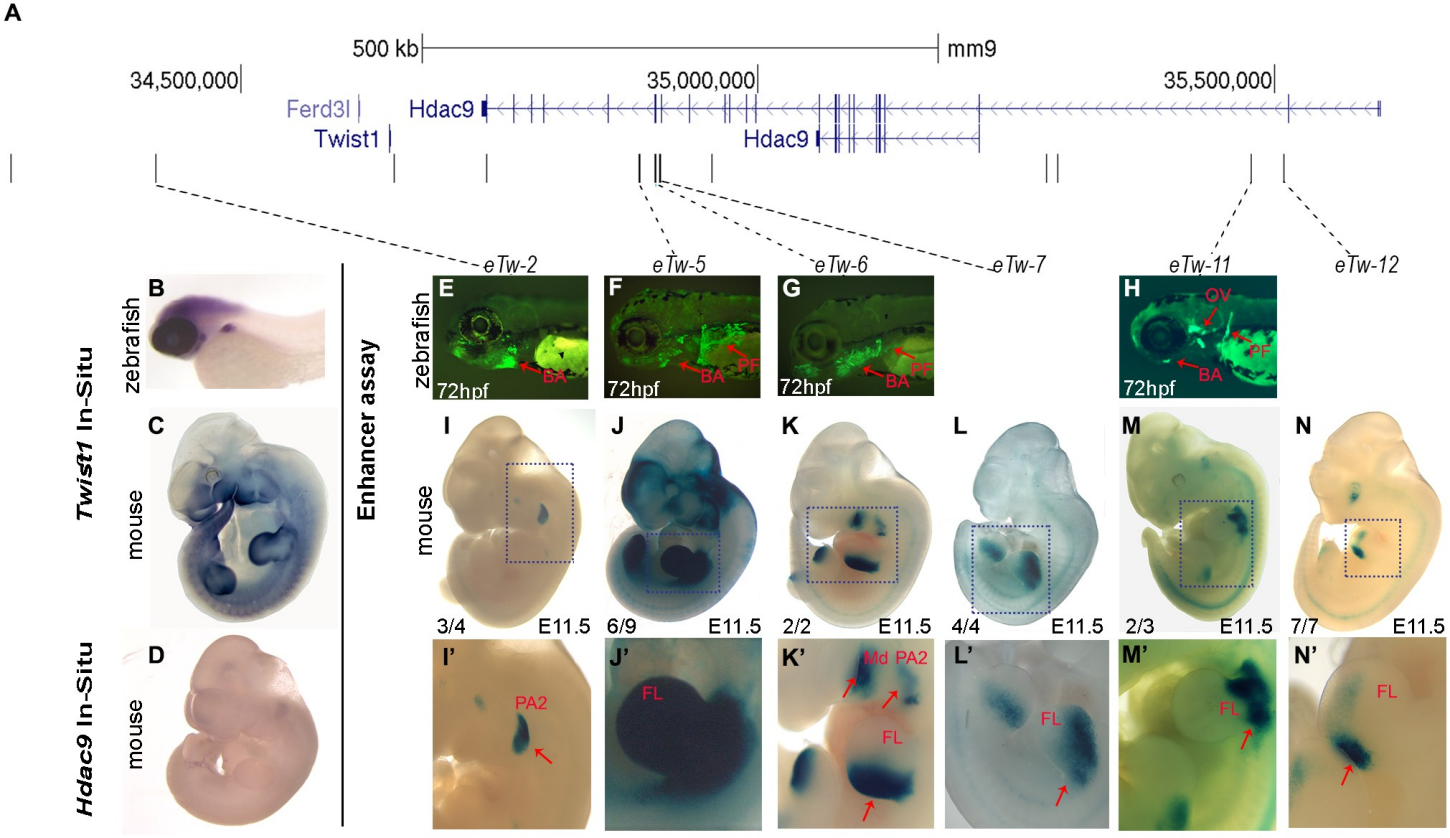
Functional enhancers in the *HDAC9-TWIST1* locus characterized using zebrafish and mice. **(A)** A scheme of the *HDAC9-TWIST1* locus. UCSC genome browser track represents genes and black bars represents enhancer candidate sequences. **(B)** Zebrafish whole-mount *in situ* hybridization of *twist1b* shows head, pectoral fin and branchial arch expression at 72 hpf. **(C)** Mouse whole-mount *in situ* hybridization of *Twist1* shows limb and branchial arch expression along the anterior-posterior axes at E11.5. **(D)** Mouse whole-mount *in situ* hybridization of *Hdac9* shows no expression in the limb and branchial arch at E11.5. **(E–H)** Tissue-specific enhancers in zebrafish embryos at 72 hpf. Tissue-specific GFP expression is indicated by red arrows: **(E)** branchial arch; **(F)** pectoral fin and branchial arch; **(G)** pectoral fin and branchial arch; **(H)** pectoral fin, otic vesicle and branchial arch. **(I-N)** Tissue-specific enhancers in mouse E11.5 embryos. Tissue-specific LacZ expression is indicated by red arrows: **(I, I')** pharyngeal arch 2; **(J, J')** whole limb bud; **(K, K')** posterior limb bud and branchial arch (known as HDAC eExon19); **(L, L')** dorsal and anterior limb bud (known as HDAC eExon18); **(M, M')** the base of the limb bud; **(N, N')** distal and posterior limb bud. The dashed boxes highlight tissue-specific expression. The numbers in the bottom left of the embryo pictures indicate the number of embryos showing this expression pattern from the total number of LacZ-stained embryos. Abbreviations: Pectoral fin (PF), branchial arch (BA), otic vesicle (OV), forelimb (FL), hindlimb (HL), mandibula (Md), pharyngeal arch 2 (PA2). Images K and L were taken with permission from Birnbaum et al. [4].

To determine the functionality of the 12 enhancer candidates, we assessed their regulatory activity using a zebrafish enhancer assay (S1 Table). Since limb and fin development are considered highly comparable at the molecular level [29], we tested fin enhancer activity during zebrafish development. Human sequences were cloned into a zebrafish enhancer vector containing the E1b minimal promoter followed by the green fluorescent protein (GFP) reporter gene [30]. These vectors were microinjected into one-cell stage zebrafish embryos along with the *Tol2* transposase for genomic integration [31]. GFP activity was monitored 48 and 72 hours post-fertilization (hpf) and compared with the known *twist1b* expression pattern (S2 Table). Six enhancer candidates drove GFP expression in specific tissues. Specifically, four enhancers (*eTw-5*, *6*, *8*, *11*) drove GFP expression in the pectoral fins, two enhancers (*eTw-*1, 5) drove GFP expression in the caudal fins, five enhancers (*eTw-2*, *5*, *6*, *8*, *11*) drove GFP expression in the branchial arches, one enhancer (*eTw-11*) drove GFP expression in the otic vesicle and one enhancer (*eTw-5*) drove GFP in somitic muscles (Fig 2, S2 Fig). Combined, we characterized six positive zebrafish enhancers that likely regulate *Twist1* fin/limb and branchial expression during embryonic development.

### Identifying active enhancers *in vivo* using a mouse enhancer assay

To characterize the activity of the zebrafish-positive enhancers in mammals, selected enhancers were chosen for transgenic enhancer assays in mice (S1 Table). The enhancer activity of three of these sequences (*eTw-5*, *6*, *7*) were previously characterized in mouse. *eTw-5* showed enhancer activity in the whole limb bud (Fig 2J and 2J'), *eTw-6*, also known as *HDAC9* eExon 19, showed enhancer activity in the branchial arch and the posterior limb bud (Fig 2K and 2K'), and *eTw-7*, known as *HDAC9* eExon 18, showed enhancer activity in the dorsal and anterior parts of the limb bud (Fig 2L and 2L') [4, 32].

We selected seven additional human sequences corresponding to the zebrafish enhancers and cloned them into the hsp68-LacZ vector that contains the heat shock protein 68 minimal promoter followed by the LacZ reporter gene [33]. While four sequences (*eTw-1*, *8*, *9*, *10*) showed no enhancer activity in mouse E11.5 embryos (S3 Fig), we found three active enhancers that presented expression patterns resembling that of *Twist1* during development (Fig 2, S3 Fig). *eTw-2* drove LacZ expression in the branchial arch (Fig 2I and 2I'), *eTw-11* drove LacZ expression in the proximal part of the limb bud where the ulna and radius are initiated (Fig 2M and 2M'), and *eTw-12* drove LacZ expression in the distal and posterior parts of the limb bud (Fig 2N and 2N').

Interestingly, the six positive enhancers presented expression patterns with partial overlap in terms of embryo developmental stages and tissues, which together resemble *Twist1* expression. Furthermore, *Hdac9* was not expressed in the limb bud or branchial arch in mouse E11.5 embryos, indicating that these enhancers do not regulate *Hdac9* in these tissues (Fig 2D). Thus, the *TWIST1-HDAC9* locus contains limb- and craniofacial-specific enhancers that likely regulate the spatiotemporal expression of *TWIST1* during development (S1 Table).

### Defining the minimal enhancer sequences required for tissue-specific activity

**Fig 3:**
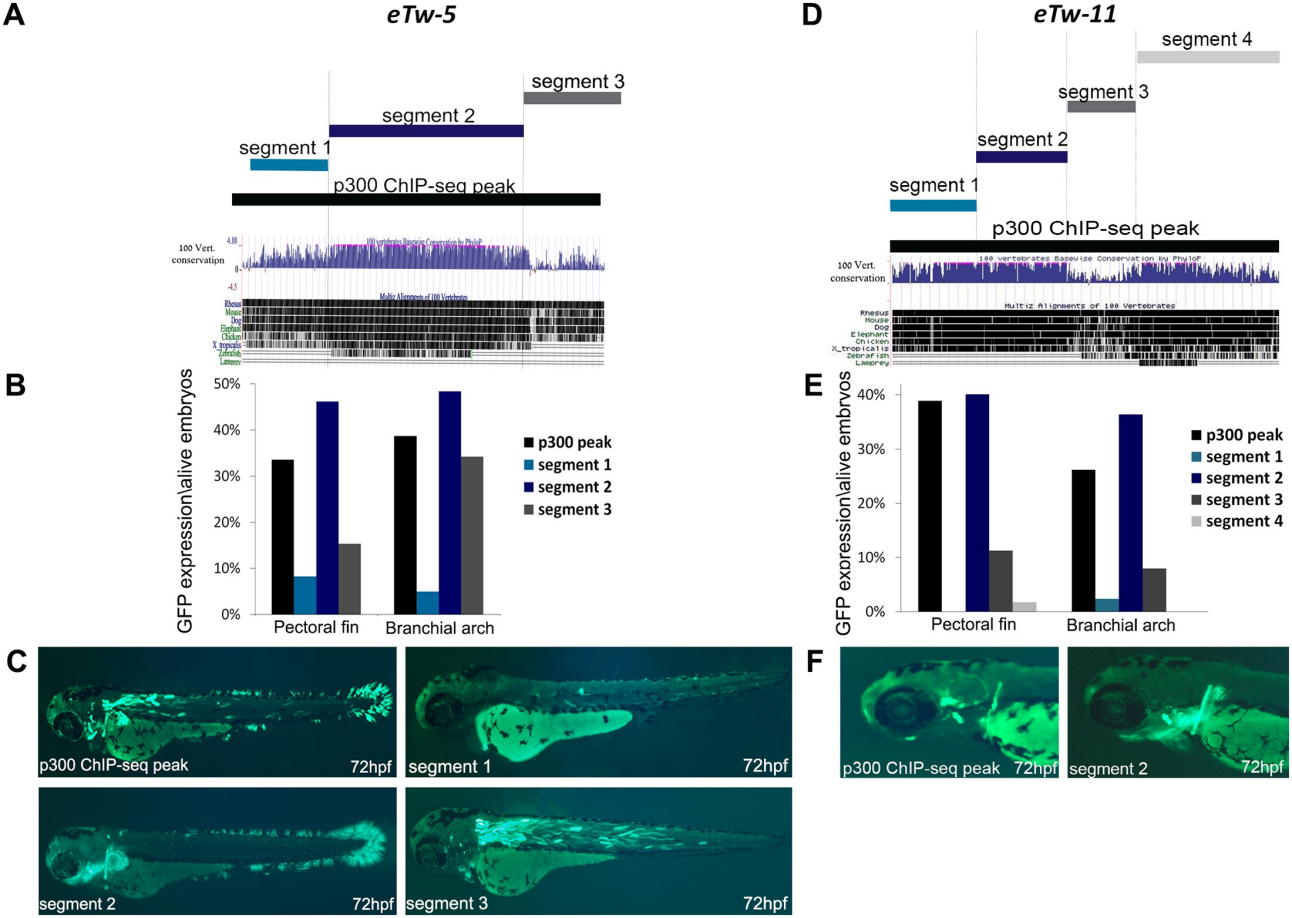
Segmental analysis of *eTw-5* and *eTw-11* enhancers in zebrafish embryos at 72 hpf. **(A)** *eTw-5* was divided into three overlapping segments: Segment 1, segment 2 and segment 3. The UCSC genome browser (http://genome.ucsc.edu) conservation track shows that segment 2 is most conserved between humans and fish. **(B)** A graph displaying the number of embryos presenting GFP expression in the pectoral fin and branchial arch tissues from all live embryos at 72 hpf. **(C)** Zebrafish enhancer assay results for *eTw-5* segments. The full sequence of the p300 ChIP-seq peak drove GFP expression in the pectoral and caudal fins, branchial arch and somitic muscles. Segment 1 did not drive GFP expression, segment 2 drove GFP expression in the fins and branchial arch and segment 3 drove GFP expression mostly in somitic muscles and to some extent in the branchial arch. **(D)** *eTw-11* was divided into four overlapping segments (segments 1–4). The UCSC genome browser (http://genome.ucsc.edu) conservation track shows that segment 3, 4 are conserved from zebrafish to humans. **(E)** A graph displaying the number of embryos with GFP expression in the pectoral fin and branchial arch tissues from all live embryos at 72 hpf. **(F)** Zebrafish enhancer assay results for *eTw-11* segments: the full sequence of the p300 ChIP-seq peak drove GFP expression in the base of the pectoral fin, in the otic vesicle and branchial arch. Segments 1, 3 and 4 did not drive GFP expression, while segment 2 drove dominant GFP expression in the base of the pectoral fin and the branchial arch.

To better characterize the functionally important sequences within the *TWIST1* enhancers, we tested the minimal sequences required for the activity of the identified enhancers in a series of segmental sequence analyses using the zebrafish enhancer assay. We selected the four positive pectoral fin enhancers *eTw-5*, *eTw-6*, *eTw-8*, *eTw-11* and analyzed their minimal enhancer sequences. *eTw-5*, which showed strong activity in the pectoral fin/limb, branchial arch and somitic muscles, was divided into three segments based on conservation level, with segment 2 harboring the most evolutionarily conserved sequence (Fig 3A). Using the zebrafish enhancer assay, we tested the enhancer activity of each segment during zebrafish development at 48 and 72 hours post-fertilization (hpf) (Fig 3B). Whereas segment 1 was not active in either the pectoral fin or the branchial arch, segment 2 showed strong activity in the pectoral fin, caudal fin and branchial arch, and segment 3 showed specific enhancer activity in the somitic muscle (Fig 3C). Segments 1 and 2 together showed similar expression patterns as segment 2, while segment 2 and 3 together showed the same activity as did the p300 ChIP-seq peak sequence (S2 Table). To further characterize the minimal enhancer sequence, we divided segment 2 into two additional segments (S4 Fig). Segment 2a drove strong epidermis GFP expression in the fish head that was not observed with intact segment 2. Segment 2b drove dominant GFP expression in both the caudal and pectoral fins but not in the branchial arch (S4 Fig). These results indicate that neither segment 2a nor 2b are sufficient for recreating the dominant pectoral fin and branchial arch expression of intact segment 2. However, segment 2a had dominant epidermis expression that was not seen in segment 2, while segment 2b exclusively resembled the caudal fin expression of segment 2 (S4 Fig). Thus, our results demonstrate that evolutionarily-conserved segment 2 is the minimal enhancer necessary for the pectoral fin- and branchial arch-specific expression.

The minimal functional sequence of the *eTw-6* enhancer was previously shown to drive strong activity in the pectoral fin/limb and the branchial arch [4]. These earlier findings facilitated further dissection of the *eTw-6* enhancer sequence. *eTw-6* was divided into four segments, classified according to evolutionary conservation and predicted binding sites of limb-expressed TFs (S5 Fig). While segments 1 and 4 were not active, segments 2 and 3 showed strong activity in specific neurons near the eyes that project into the trunk (S5B Fig) but no pectoral fin or branchial arch activity. Thus, *eTw-6* sequence is required for activating pectoral fin and brachial arch expression and encompasses *HDAC9* coding exon 19 and its 5' intron.

*eTw-8*, which showed strong activity in the pectoral fin and branchial arch, was divided into three segments based on the level of conservation, with segment 3 being the most conserved sequence (S6A Fig). While segments 1 and 2 were not active in either the pectoral fin or the branchial arch, segment 3 showed activity in both tissues (S6B and S6C Fig), thus demonstrating that the evolutionary-conserved segment 3 is sufficient to drive branchial arch expression.

*eTw-11*, which showed strong activity in the base of the pectoral fin, in the otic vesicle and in the branchial arch, was divided into four segments based on conservation level and predicted binding of limb-expressed TFs (Fig 3D). Segments 1, 3 and 4 were not active in the base of the pectoral fin, in the otic vesicle or in the branchial arch. However, segment 2 showed specific activity in both the pectoral fin and branchial arch, as seen with the p300 ChIP-seq sequence (Fig 3E and 3F). Interestingly, GFP expression in the base of the pectoral fin resembled the specific limb-bud activity of this enhancer in mouse E11.5 embryos (Fig 2M and 2M'). Thus, *eTw-11* segment 2 is the minimal enhancer required for the pectoral fin and branchial arch expression.

### The *Twist1* promoter interacts with the characterized limb and branchial arch enhancers

**Fig 4:**
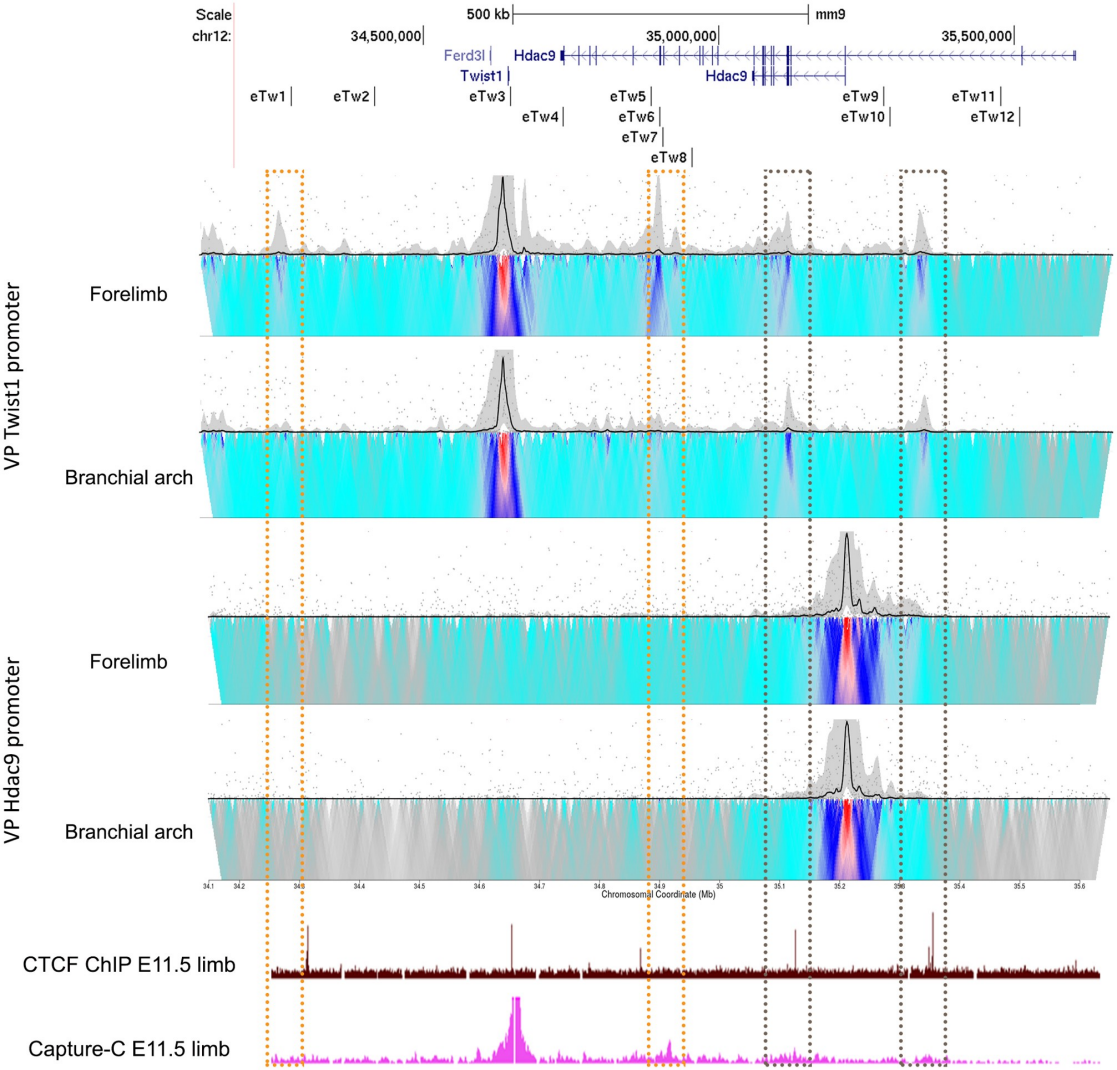
Chromatin interactions in the *Twist1-Hdac9* locus. 4C-seq interaction profiles of *Twist1* and *Hdac9* promoters as viewpoints in the forelimb and branchial arch of mouse E11.5. Limb-specific interacting regions of the *Twist1* promoter are presented in the orange dashed box. Interacting regions of the *Twist1* promoter in the limb bud and branchial arch that overlap with CTCF-occupied sites are presented in the gray dash boxes. UCSC genome browser tracks of CTCF ChIP-seq and Capture-C from the mouse E11.5 limb bud [27].

To assess whether the characterized enhancers regulate *Twist1* expression, we elucidated their physical interactions with the *Twist1* promoter. We performed circularized chromosome conformation capture (4C) coupled with next-generation sequencing (4C-seq) experiments in mouse E11.5 limb buds and branchial arches using *Twist1* and *Hdac9* promoters as viewpoints. *Twist1* promoter showed high frequency interactions with a region that contains a cluster of three enhancers (*eTw-5*, *6 and 7*) in the limb bud and low frequency interactions in the branchial arch (Fig 4). Moreover, *Twist1* promoter region that also contains CTCF site showed high frequency interactions with two regions overlapping CTCF sites in both limb buds and the branchial arch (Fig 4). Interestingly, while the *Twist1-Hdac9* locus contains dozen of CTCF sites, these two interacting regions are occupied by CTCF in the limb bud at E11.5 [27], indicating that these CTCF sites are likely involved in the chromatin looping of the *Twist1* locus. In contrast, the 4C-seq results for the *Hdac9* promoter region did not reveal interactions with any region in either limb buds or branchial arch tissues (Fig 4). Thus, *Hdac9* coding and non-coding sequences encompass enhancers and CTCF sites that are found in physical proximity to the *Twist1* promoter region of mouse E11.5 limb bud and branchial arch.

### LMX1B and TFAP2 regulate the activity of *TWIST1* enhancers

**Fig 5:**
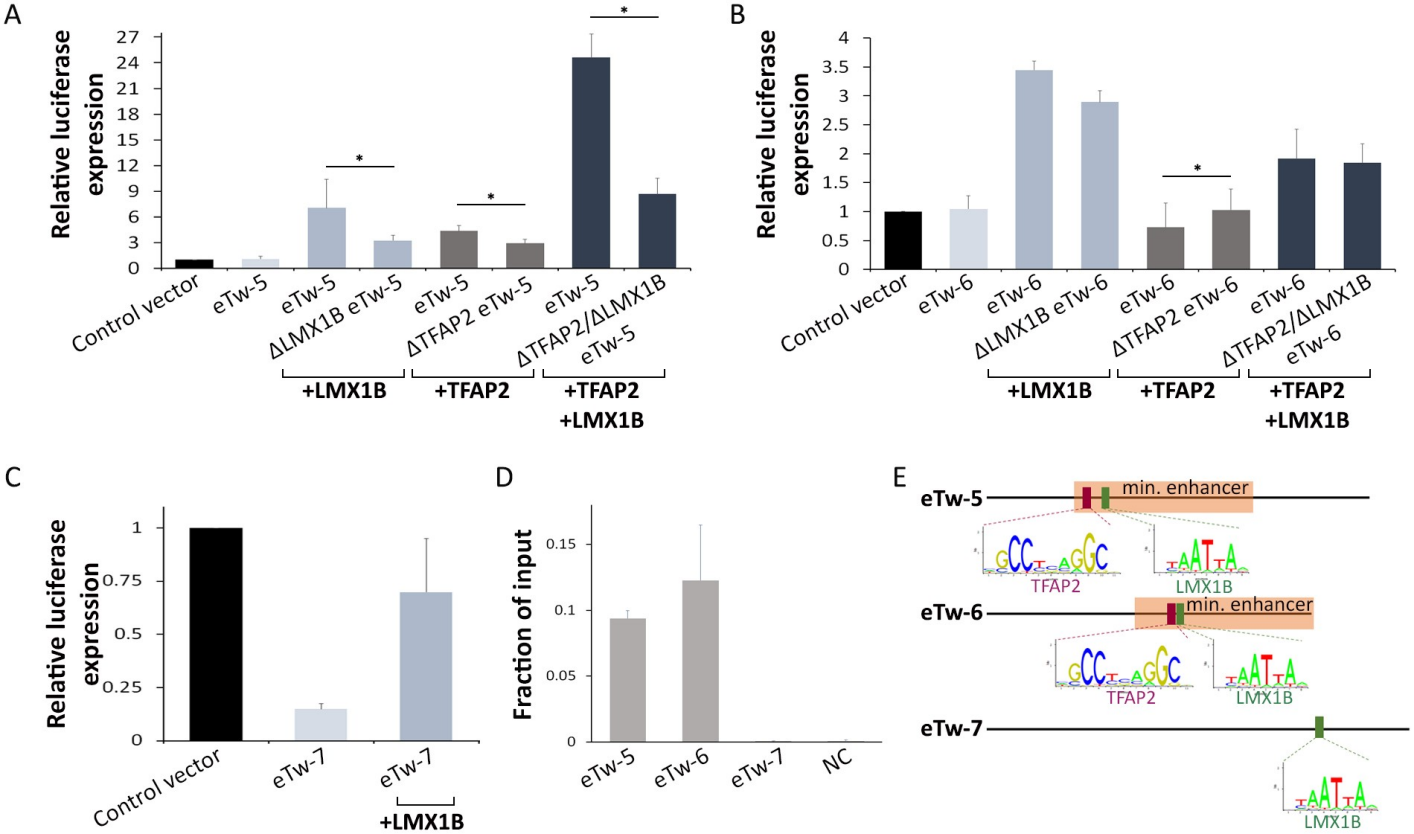
LMX1B and TFAP2 modulate *TWIST1* enhancers. **(A)** *In vitro eTw-5* activity in HEK293T cells following co-transfection with LMX1B or TFAP2 (TFAP2α and/or TFAP2γ) expression vectors. Luciferase activity was measured for the *eTw-5* sequence and for deleted LMX1B (ΔLMX1B), TFAP2 (ΔTFAP2) or both LMX1B and TFAP2 (ΔLMX1B\ΔTFAP2) binding sites from *eTw-5* sequence. **(B)** *In vitro eTw-6* activity in HEK293T cells following co-transfection with LMX1B or TFAP2 (TFAP2α and/or TFAP2γ) expression vectors. Luciferase activity was measured for the *eTw-6* sequence and deleted LMX1B (ΔLMX1B), TFAP2 (ΔTFAP2) or both LMX1B and TFAP2 (ΔLMX1B\ΔTFAP2) binding sites from *eTw-6* sequence. **(C)** *In vitro eTw-7* activity in HEK293T cells following co-transfection with LMX1B expression vector. Luciferase activity was measured for the *eTw-7* sequence and for LMX1B-deleted *eTw-7* (ΔLMX1B). Relative luciferase expression results after normalization to Renilla activity are presented and represent the means ± standard deviation of three independent experiments (P-value<0.05, Student’s t-test). **(D)** ChIP-qPCR analysis of *eTw5-7* with anti-TFAP2α antibodies in a mouse E11.5 limb bud. Fold enrichment is presented as a fraction of input. The error bars represent the SD from two technical replicates of a representative experiment. **(E)** A schematic representation of minimal enhancer sequences with the location of the predicted binding site for TFAP2 (red bar) and LMX1B (green bar) shown.

We next sought to assess the distinct transcription factor repertoire required for *TWIST1* enhancer activity. We analyzed *eTw*-5, 6 and 7 sequences for predicted transcription factor-binding sites using TRANSFAC [34] and JASPAR [35] and identified putative binding sites of six limb-expressed TFs: HAND2, HOXD13, TFAP2α and TFAP2γ, LMX1B, and MSX2. To experimentally test these predictions, we used the luciferase reporter assay in HEK293T cells that were co-transfected by these three enhancers and the relevant TF expression vector. Whereas co-transfection with the enhancers and Hand2, Hoxd13 or MSX2 expression vectors showed no significant effect on enhancer activity, co-transfection with LMX1B and/or TFAP2α and TFAP2γ (termed TFAP2 when both TFs were introduced) expression vectors modulated the activity of the three enhancers (Fig 5). Specifically, *eTw-5* enhancer activity was increased 7-fold upon co-transfection with LMX1B expression vector and 4.5-fold with TFAP2 expression vector (Fig 5A). Remarkably, a synergistic 24-fold effect was observed upon co-transfection of both LMX1B and TFAP2 expression vectors with *eTw5* (Fig 5A). Co-transfection of LMX1B expression vector along with *eTw-5* which was mutated at the predicted LMX1B-binding site (ΔLMX1B eTw5) showed 50% reduced activity. Similarly, co-transfection of TFAP2 expression vector with a *eTw-5* mutated in the TFAP2-binding site (ΔTFAP2 eTw5) showed 30% reduced activity (relative to the wild type *eTw-5* sequence). Moreover, when both the LMX1B- and TFAP2-binding sites were mutated (ΔTFAP2\LMX1B eTw5), we observed a 65% reduction in *eTw-5* activity, suggesting an additive effect of these TFs on *eTw-5* activity (Fig 5A).

*eTw-6* enhancer activity was increased 3.5-fold upon co-transfection with the LMX1B expression vector (Fig 5B). However, *eTw-6* enhancer activity was reduced upon co-transfection with a TFAP2 expression vector (Fig 5B). Notably, co-transfection with both LMX1B- and TFAP2 expression vectors led to two-fold increase in enhancer activity, as compared to the control empty vector (Fig 5B), suggesting a combined effect of LMX1B activation and TFAP2 repression. Furthermore, *eTw-6* mutated in the LMX1B-binding site (ΔLMX1B eTw6) revealed only 15% reduced activity (P = 0.053, student’s t-test), and *eTw-6* mutated in the TFAP2-binding site (ΔTFAP2 eTw6) showed 40% increased activity, relative to wild type *eTw-6* sequence. As expected, a double mutation affecting both the LMX1B- and TFAP2-binding sites (ΔTFAP2\LMX1B eTw6) did not have a significant effect on *eTw-6* activity (Fig 5B), thus further supporting an antagonistic effect of these two factors. Finally, the basal activity of *eTw-7* was lower than that with the empty vector in this cell line. However, upon co-transfection of *eTw-7* with the LMX1B expression vector, we observed a significant elevation in enhancer activity (Fig 5C) that was not seen with the TFAP2 expression vector, which has no binding site in the *eTw-7* enhancer sequence. Notably, we tested the *in vivo* effect of LMX1B and TFAP2 binding site mutants on *eTw-5* and *eTw-6* enhancer activity using zebrafish enhancer assay. Zebrafish embryos were microinjected with enhancer vectors containing *eTw-5* and *eTw-6* sequences mutated for LMX1B binding sites that showed a significant decrease in embryos expressing GFP in the pectoral fin (9% and 0%, respectively) compared to those with control enhancer sequences (46% and 21%, respectively; P<0.05, chi square test). These results demonstrate the importance of LMX1B binding sites for the activity of both enhancers (S2 Table). Furthermore, zebrafish embryos that were microinjected with enhancer vectors containing *eTw-5* and *eTw-6* sequences mutated for TFAP2 binding sites showed an increase in embryos expressing GFP in the branchial arch (78% and 91%, respectively), and no change in embryos expressing GFP in the pectoral fin, compared to control enhancer sequences (48% and 78%, respectively) (S2 Table).

Thus, LMX1B and TFAP2 serve as excellent candidates for modulating the activity of *TWIST1* enhancers. Specifically, while LMX1B induced the activity of the three selected enhancers, TFAP2 induced *eTw-5* and repressed *eTw-6* activity in this assay.

### Tfap2- and Lmx1B-occupied *Twist1* enhancers in the limb bud

To validate the roles of Lmx1b and Tfap2 on endogenous *Twist1* enhancer activity, we tested the TF occupancy on the enhancer sequences in mouse E11.5 limb buds. We carried out ChIP-qPCR with anti-Tfap2 antibodies and found high enrichment of Tfap2 occupancy on *eTw-5* and *eTw-6* enhancers in the limb bud of E11.5 mouse embryos but not on the *eTw-7* enhancer or the negative control (which both lack a Tfap2-binding site) (Fig 5D). Furthermore, analysis of previously reported ChIP-seq data generated with anti-Lmx1b antibodies on mouse E11.5 limb showed high enrichment of three enhancers that contain Lmx1b-binding sites [36]. Thus, both Lmx1b and Tfap2 bind the enhancers of *Twist1 in vivo*, and hence likely play a role in regulating their activities.

### Deletion of *eTw5-7* reduces *Twist1* expression and leads to PPD

**Fig 6:**
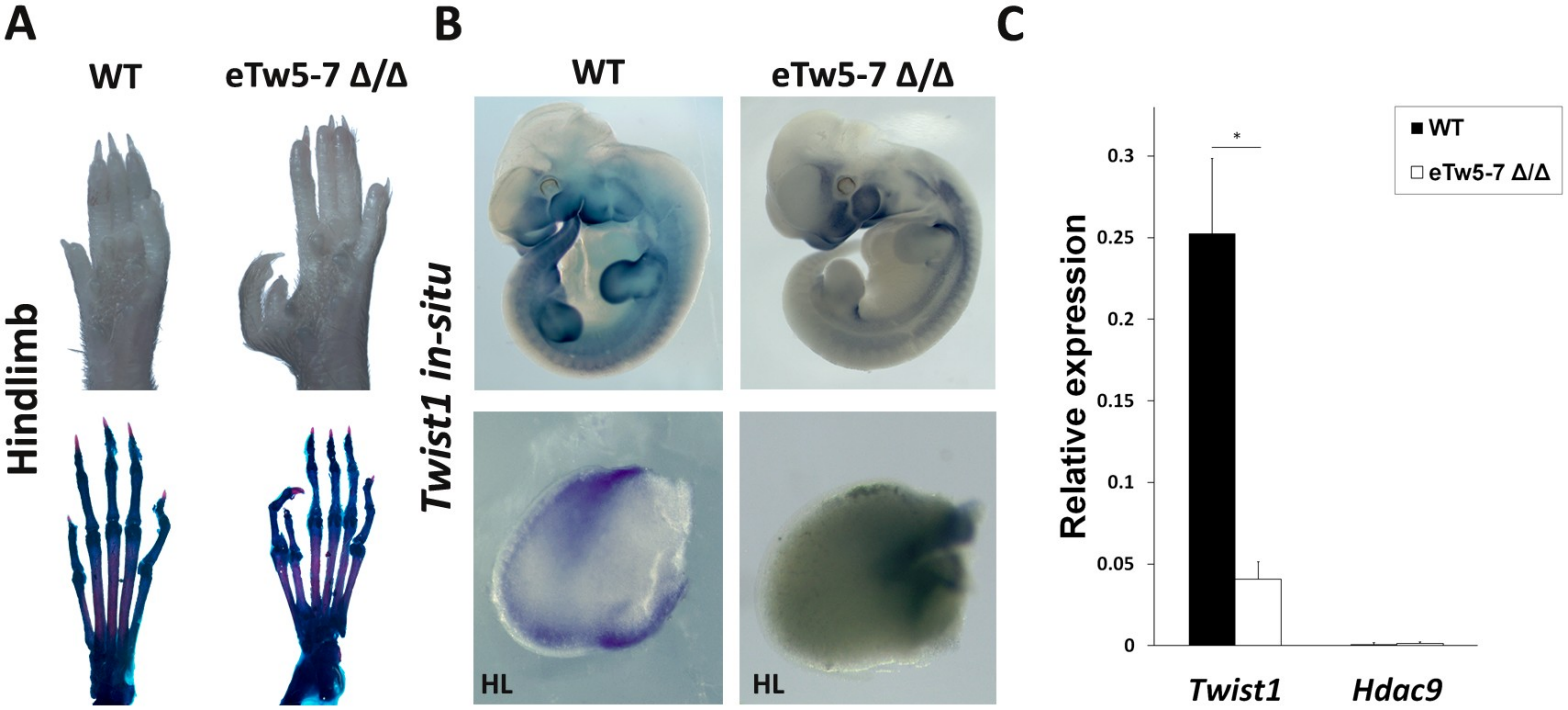
Reduced *Twist1* limb expression and polydactyly in *eTw5-7*^Δ\Δ^. **(A)** Hindlimb autopods and corresponding skeletal preparations of wild type (WT) and eTw5-7^**Δ\Δ**^ mice. Pre-axial polydactyly is presented in *eTw5-7*^***Δ\Δ***^ mice. **(B)** Top: mouse whole mount *in situ* hybridization of *Twist1* in WT and *eTw5-7*^***Δ\Δ***^ mice at E11.5. Bottom: Reduced *Twist1* expression in E11.5 limb bud along the anterior-posterior axes in *eTw5-7*^***Δ\Δ***^. **(C)** Expression levels of *Twist1* and *Hdac9* in the mouse E11.5 hindlimb of wild type and *eTw5-7*^***Δ\Δ***^ mice measured by quantitative RT-PCR (p>0.05, Student’s t-test).

To evaluate the *in vivo* effect of *Twist1* enhancers on limb development, we generated a 23 kb sequence deletion of *eTw5-7* enhancers in mice using CRISPR/Cas9 genome editing. Notably, such deletion encompassed all three enhancers and exons 17–20 of *Hdac9* (23,137bp; chr12:34883772–34906909; mm9). As all three enhancers have overlapping activities and interacted with the *Twist1* promoter region, we tested the effects of deletion of all three enhancers on *Twist1* transcription regulation. Although both heterozygous and homozygous *eTw5-7* deletion mice were viable and showed the expected Mendelian inheritance ratios, homozygous *eTw5-7* deletion mice (*eTw*5-7^Δ\Δ^) displayed a preaxial polydactyly (PPD) phenotype (Fig 6). We found that 79 of 103 (77%) of the *eTw5-7*^*Δ\Δ*^ mice displayed hindlimb polydactyly and 45 of 103 (44%) of these mice displayed forelimb polydactyly, indicating partial penetrance with variable expression in *eTw5-7*^*Δ\Δ*^ mice.

We next evaluated the effect of *eTw5-7* deletion on *Twist1* expression. Using whole mount *in situ* hybridization, we showed that *Twist1* expression is reduced in the limb bud of eTw5-7^Δ\Δ^ E11.5 mouse embryos (Fig 6B). Using quantitative RT-PCR, we found 5-fold reduced expression (P<0.05) of *Twist1* in the hindlimb of *eTw5-7*^*Δ\Δ*^ mice (Fig 6C). Notably, *Hdac9* is neither expressed in wild type nor in *eTw5-7*^*Δ\Δ*^ mice, thus excluding its impact on the observed phenotype. Taken together, our data indicate that disruption of *eTw5-7* enhancers leads to reduced *Twist1* limb bud expression and PPD, which mimics the PPD phenotype previously observed in a *Twist1*^+\-^ mouse model.

## Discussion

In this study, we characterized transcriptional enhancers that regulate spatiotemporal *Twist1* activity during limb and craniofacial development. We identified 12 enhancer candidates located in the *HDAC9-TWIST1* synteny block and characterized their expression patterns using transgenic zebrafish and mice. Of the 12 enhancer candidates, we identified 8 active enhancers, each having a discrete activity pattern that recapitulated aspects of the *Twist1* expression pattern during development of zebrafish and mice. The partial overlapping activity pattern of the analyzed *Twist1* enhancers might ensure robustness of *Twist1* expression during development. For example, *eTw-5*, *eTw-6* and *eTw-12*, are active enhancers in the posterior limb bud, while *eTw-5*, *eTw-7* and *eTw-11* enhancers are active in the anterior and dorsal proximal limb bud, indicating that the discrete activity of each enhancer, along with the overlapping activity between enhancers, is essential for proper spatiotemporal *Twist1* expression. Indeed, deletion of *eTw5-7* enhancers leads to reduced *Twist1* limb expression and caused PPD.

This PPD phenotype in *eTw5-7*^*Δ\Δ*^ is dominant in the hindlimb, which is similar to the *Twist1*^+\-^ PPD phenotype (42%), yet with higher penetrance (77%). This deletion also led to PPD in the forelimb with lower penetrance (44%). The differential expression and partial penetrance of PPD is consistent with altered *Twist1* transcription regulation. While *eTw-6* and *eTw-7* have discrete activities in the posterior and anterior limb buds, respectively, both enhancers have partial overlapping activity with the *eTw-5* limb bud enhancer, indicating that the loss of enhancer activity along the anterior-posterior axis of *eTw5-7*^*Δ\Δ*^ limb bud leads to reduction of *Twist1* limb expression and to a PPD phenotype. Together, the enhancers characterized in this study generate a *Twist1* transcriptional regulatory network that plays a role in limb development.

Previous studies have shown that human enhancer sequences can function as active enhancers in zebrafish, even without homologous sequences in zebrafish [4]. Our results support these findings for most of the enhancers considered. For example, *eTw-2* and *eTw-11*, which do not have homologous sequences in zebrafish, show similar enhancer expression patterns in zebrafish and mice (Fig 2E, 2I, 2H and 2M). However, *eTw-1* and *eTw-8* show fin expression in zebrafish, yet did not show limb expression in mice (S2A and S2E Fig). At the same time, *eTw-7* and *eTw-12* did not show fin expression but were positive limb enhancers (Fig 2L and 2N). These discrepancies between fin and limb enhancer activity could be attributed to differences in fin versus limb development, the use of a generic minimal promoter (e.g. the e1b promoter for zebrafish and the hsp68 promoter in mice), or the possibility that these enhancer sequences were considered out of context in the *in vivo* enhancer assay. Moreover, it is likely that additional uncharacterized enhancers are located in this region. Therefore, further studies are needed to fully elucidate the regulatory mechanism of *Twist1* during development.

We sought to characterize the mechanism of action of the various enhancers. Accordingly, we first identified the minimal enhancer sequences required for activity in zebrafish. In general, there was strong correlation between evolutionary conservation and activity in the tested enhancers. As such, we showed that segment 2 of *eTw-5* promoted a specific GFP expression pattern in the pectoral fin and branchial arch, recapitulating the p300 ChIP-seq peak activity (Fig 3B and 3C). Similarly, segment 2 of *eTw-11* showed specific activity in both the pectoral fin and branchial arch, also recapitulating the p300 ChIP-seq peak activity (Fig 3E and 3F). These findings indicate that these minimal enhancer sequences show high evolutionary conservation. The minimal segments that harbor binding sites for tissue-specific expressed TFs and function as enhancers might be subject to strong negative selection to maintain their activity.

The mechanism of action of *TWIST1* enhancers involves both a TF repertoire that depends on motif-positioning and the chromatin conformation of the *HDAC9-TWIST1* locus. We tested several limb-expressed TFs (LMX1B, TFAP2 α\γ Hand2, Hoxd13, MSX2) and found that LMX1B and TFAP2 α\γ regulated the *TWIST1* enhancers (Fig 5). LMX1B is a LIM homeobox transcription factor which plays a central role in dorso-ventral patterning of the vertebrate limb and in skull formation [37]. Furthermore, LMX1B also has an unexplained indirect effect on anterior-posterior limb patterning [37]. In line with the fact that Lmx1b expression overlaps the limb-specific activity of *Twist1* enhancers, it was shown that Lmx1b occupies these *Twist1* enhancers in the limb bud and activates their enhancer function [36]. Furthermore, deletion of Lmx1b-binding sites altered the activity of these enhancers (Fig 5). These results demonstrate the role of Lmx1b in *Twist1* regulation and elucidate the indirect effect of Lmx1b on anterior-posterior limb patterning.

The transcription factors TFAP2α and TFAP2γ are expressed in the limb and branchial arch and are essential for vertebrate embryogenesis [38, 39]. TFAP2α primarily regulates the initial formation and migration of neural crest cells, a process in which TWIST1 is also essential. In the absence of Tfap2α, mouse embryos present a range of limb and craniofacial malformations [40], including polydactyly and severe dysmorphogenesis of the face and skull [41]. Absence of Tfap2γ is implicated in placental development but not in limb or craniofacial development, suggesting that Tfap2α has dominant activity over Tfap2γ in the limb and branchial arch [39]. In support of this notion, we found that TFAP2α and TFAP2γ together have an additive regulatory effect on the *eTw-5* and *eTw-6* enhancers. Moreover, we found that Tfap2α binds *eTw-5* and induces its activity, while Tfap2α binds *eTw-6* and reduces its activity. This dual function as activator and repressor affects the spatiotemporal regulation of *Twist1* expression that is required for normal limb and craniofacial development. Our study thus identified two TFs, LMX1B and TFAP2, that participate in the TF repertoire that is likely required for the activity of *TWIST1* enhancers. However, further investigation of the complete TF repertoire is required to delineate the mechanism of *TWIST1* transcriptional regulation.

The activities of the characterized enhancers reported here are supported by specific chromatin interactions of the *Twist1*-*Hdac9* locus in the limb bud and branchial arch. 4C-seq data demonstrated that the *eTw-5*, *6* and *7* enhancers interact with the *Twist1* promoter region in the limb bud, as well as in the branchial arch, demonstrating the strong *Twist1* enhancer activity of these sequences in the limb bud. Furthermore, Capture-C data that was collected on the mouse E11.5 limb bud showed that the *Twist1* promoter interacts with a distal region that encompasses the three enhancers, *eTw5-7* (Fig 4) [27]. Interestingly, the *Twist1* promoter region contains a CTCF site and interacts with two distal CTCF-occupied sites located 475 and 700 kb away that likely generate promoter-enhancer interactions that promote *Twist1* enhancer activity in the limb bud and prevent the activity of *Hdac9* promoter located between the two occupied CTCF sites. Thus, the specific chromatin looping of the *Twist1*-*Hdac9* locus in the limb bud promotes enhancer-promoter interactions that are likely essential for discrete *Twist1* expression.

In summary, we have characterized enhancers with expression overlapping that of *Twist1* during embryo development in zebrafish and mouse, and showed their essential roles in *Twist1* regulation in the developing limb. We further delineated the specific minimal enhancer sequences and the interacting TFs that play a role in their activity. Our findings pave the way for future studies that could identify mutations in regulatory elements leading to congenital limb and craniofacial malformations in humans.

## Materials and methods

### Ethics statement

All animal work was approved by the Ben Gurion. Institutional Animal Care and Use Committee protocol number 52-09-2016.

### ChIP-seq data analyses

Ten available ChIP-seq and ATAC-seq data sets that were obtained with E10.5/E11.5 mice embryos limb and branchial arch tissues and which used enhancer-associated marks (EP300, H3K27ac) were analyzed for redundancy, as well as evolutionary conservation. ChIP-seq peaks obtained from limb buds with the EP300 marker [26] served as the reference peak for overlap analysis. Overlapping peaks were defined as when at least 1 bp regions overlapped.

### ChIP-qPCR

Mouse embryos were harvested from timed pregnant C57Black females (Envigo, Indianapolis, IN) at E11.5. The limb buds were dissected, washed twice in cold phosphate-buffered saline and crosslinked with 1% formaldehyde for 10 min. Chromatin was isolated and sheared using a Bioruptor (Diagenode, Denville, NJ). Immunoprecipitation was performed using 5 mg of anti-Tfap2α antibodies (sc-12726, Santa-Cruz Biotechnology). Next, we performed quantitative PCR on targeted sequences using Syber-fast mix (Kapa Biosystems, Roche) and found specific enrichment for *Twist1* enhancers but not for randomly selected sequences (S3 Table). Chromatin from the same sample was processed for the input control.

### Transgenic enhancer assay

Primers were designed to amplify the candidate enhancer sequences from human genomic DNA (S3 Table). PCR products were cloned into the E1b-GFP-Tol2 enhancer assay vector containing an E1b minimal promoter followed by the gene for GFP [30]. These constructs were injected into zebrafish embryos using standard procedures. For statistical significance, at least 100 embryos were injected per construct in at least two different injection experiments along with Tol2 mRNA to facilitate genomic integration [31]. GFP expression was observed and annotated 48 and 72 hpf. An enhancer was considered as a positive enhancer when 30% of the live embryos showed a consistent GFP expression pattern. In the mouse enhancer assay, the same human genomic fragments used in zebrafish were cloned into a vector containing the Hsp68 minimal promoter followed by the LacZ reporter gene [33]. Transgenic mice were generated by Cyagen Biosciences using standard procedures. Embryos were harvested at E11.5 and stained for LacZ expression as described previously [33].

### Generation of *eTw5-7* deletion mice using CRISPR/Cas9

A mouse strain carrying deleted *eTw5-7* alleles were created using a modified CRISPR/Cas9 protocol [42]. Briefly, two sgRNAs targeting a 23 kb sequence that encompasses the three enhancers and exons 17–20 of Hdac9 (23,137bp; chr12:34883772–34906909; mm9) were designed using CHOPCHOP [43]. No potential off-targets were found when searching for matches in the mouse genome (mm9) when allowing for up to two mismatches in the 20 nucleotide-long sequence preceding the PAM sequence. The T7 promoter was added to the sgRNA template, and the whole cassette was chemically synthetized by IDT. The PCR amplified T7-sgRNA product was used as template for *in vitro* transcription using the MEGAshortscript T7 kit (Thermo Fisher Scientific). Cas9 mRNA was transcribed *in vitro* using the mMESSAGE mMACHINE T7 kit (Thermo Fisher Scientific). The DNA template for *in vitro* transcription containing the humanized *Streptococcus pyogenes* Cas9 gene was PCR-amplified from the px330 plasmid. *eTw5-7* deletion mice were generated by injecting a mix of Cas9 mRNA (final concentration of 100 ng/ul) and sgRNA (50 ng/ul) in injection buffer (10 mM Tris, pH 7.5; 0.1 mM EDTA) into the cytoplasm of C57black embryos in accordance with standard procedure approved by Ben-Gurion University. Female mice of the ICR (CD-1) strain were used as foster mothers. F0 mice were genotyped using PCR to detect deletion of enhancers from the mouse genome (S7 Fig, S3 Table).

### 4c-seq

Limb bud and branchial arch tissues of mouse E11.5 embryos were micro-dissected and dissociated into single cells that were fixed with 2% formaldehyde. Nuclear DNA was digested using DpnII and BfaI (4ure bp cutters) as primary and secondary restriction enzymes, respectively, followed by ligation using T4 DNA ligase [44]. Ligated fragments were amplified by inverse PCR for each viewpoint (for primer sequences, see S3 Table). PCR products were sequenced with a 50 bp single-end read on an Illumina HiSeq machine. Sequenced reads were split into libraries based on their barcodes, and then by their primer (P1 or P2 for each library), resulting in about 1.5 million reads per library/primer. Bait sequences were trimmed and the remaining sequences were mapped to the mouse genome (mm9, limited to chr12) using Bowtie [45]. In addition, we used 4Cseqpipe [44] to generate 4C-seq contact profiles. The 4C-seq raw data deposit in GEO accession GSE116821.

### Cell culture and reporter assays

HEK293T cells (1–2x10^5^) were cultured in 24-well plates using standard protocol [46]. Cells were transfected with 500 ng of the pGL4.23 plasmid cloned with the enhancer candidate, along with 10 ng of Renilla, using PolyJet transfection reagent (SignaGen). After 24 h, enhancer activity was measured using the Dual-Luciferase reporter assay (Promega) on a SPARK microplate reader (Tecan). In HEK293T cells, the enhancer variant vectors were co-transfected along with expression constructs (100 ng) for LMX1B (obtained from Brendan Lee) [47], SP(RSV)-TFAP2α (obtained from Trevor Williams) [48], CMX-TFAP2γ (obtained from Hubert Schorle) [49], or MSX2 (from Sylvie Babajko) [50] or plasmids pcDNA3-1Xflag-Hand2 or pCMV5-Hoxd13 (from Rolf Zeller) [51] to measure the effect of each construct on enhancer activity. Both TFAP2α and TFAP2γ had effects on enhancer activity but when transfected together their effect was significantly higher. We also generated a series of deletions in the LMX1B- and TFAP2-binding sites of *eTw5-7* by site-directed mutagenesis (for primer sequences, see S3 Table). As a positive control for luciferase activation, we used 500 ng of pGL4.13 plasmid (Promega) that contains an AP-1 responsive element.

### Whole-mount *in situ* hybridization

Zebrafish embryos were collected from wild type matings between 24 and 72 hpf and fixed in 4% paraformaldehyde. A full-length zebrafish twist1b (gifted by Prof. Ina Gitelman) cDNA clone was used to generate a digoxygenin-labeled probe. Whole-mount *in situ* hybridizations were performed according to standard protocols [52]. Mouse E11.5 embryos were fixed in 4% paraformaldehyde. Clones containing mouse Twist1 [53] and Hdac9 (EMM1032-601163 and EMM1002-6974502, Open Biosystems) were used as templates with the digoxygenin-labeled probes. Mouse whole-mount *in situ* hybridizations were also performed according to standard procedures [54].

## Supporting information

S1 Table List of enhancer candidates in the *TWIST1-HDAC9* locus. (XLSX)

S2 Table Summary of enhancer assay in zebrafish. (XLSX)

S3 Table Primer list. (XLSX)

S1 Fig Analysis of TAD boundaries in the *Twist1-Hdac9* locus. **(A-C)** Heat map representing the chromatin interaction frequencies from Hi-C data at the *Twist1-Hdac9* locus in: **(A)** mouse embryonic stem cells (mESC) [22], **(B)** mouse cortex [22], and **(C)** mouse erythroleukemia CH12-LX [23]. TAD boundaries analysis by PSYCHIC for **(D)** mouse embryonic stem cells (mESC) [22], **(E)** mouse cortex [22], and **(F)** mouse erythroleukemia CH12-LX [23]. The *Twist1* and *Hdac9* genes reside in the same TAD across the different cell types. Each column represents the interaction intensity in a 50 kb window.
(TIF)

S2 Fig Functional enhancers in the *HDAC9-TWIST1* locus characterized using zebrafish. **(A)** *eTw-1* drives GFP expression in the caudal fin. **(B)** *eTw-2* drives GFP expression in the branchial arch. **(C)** *eTw-5* drives GFP expression in the pectoral fin, branchial arch and somitic muscles. **(D)** *eTw-6* drives GFP in the pectoral fin and branchial arch. Top: lateral view. Bottom: ventral view. **(E)** *eTw-8* drives GFP in expression in the branchial arch. **(F)** *eTw-11* drives GFP expression in the base of the pectoral fin, in the branchial arch and in the otic vesicle. Top: lateral view. Bottom: dorsal view.
(TIF)

S3 Fig Identification of functional enhancers in the *HDAC9-TWIST1* locus using a mouse enhancer assay. **(A)** *eTw-1* does not show LacZ expression. **(B, C)** *eTw-2* drives LacZ expression in the branchial arch. **(D)** *eTw-8* does not show LacZ expression. **(E)** *eTw-11* drives LacZ expression in the branchial arch and limb buds. **(F-H)** *eTw-12* drives LacZ expression in the anterior limb bud. Pharyngeal arch 2 (PA2), Mandibula (Md), Forelimb (FL).
(TIF)

S4 Fig Additional segmental analysis of *eTw-5* enhancer function in zebrafish embryos at 72 hpf. **(A)** The *eTw-5* segment 2 was divided into segments 2a and 2b. **(B)** A graph displaying the number of embryos presenting GFP expression in the pectoral and caudal fins and branchial arch out of all live embryos at 72 hpf. **(C)** Zebrafish enhancer assay results for *eTw-5* segments: segment 2a drives GFP expression in the epidermis surrounding the head and segment 2b drives GFP expression in the pectoral and caudal fins.
(TIF)

S5 Fig Segmental analysis of *eTw-6* enhancer function in zebrafish embryos at 72 hpf. **(A)** The *eTw-6* enhancer that drives GFP expression in the pectoral and branchial arch was divided into four overlapping segments (1–4). The UCSC genome browser (http://genome.ucsc.edu) conservation track shows that segment 4 which contains *Hdac9* exon 18 is conserved between humans and fish. **(B)** A graph displaying the number of embryos with GFP expression in the pectoral fin, branchial arch, brain and specific neurons out of all live embryos at 72 hpf. **(C)** Zebrafish enhancer assay results for *eTw-6* segments: segment 1 and segment 4 did not drive GFP expression, while segments 2 and segment 3 drove GFP expression in neurons near the eyes that project to the trunk.
(TIF)

S6 Fig Analysis of *eTw-8* enhancer function in zebrafish embryos at 72 hpf. **(A)** *eTw-8* was divided into three segments (1–3). The UCSC genome browser (http://genome.ucsc.edu) conservation track shows that segment 3 is the most evolutionarily conserved. **(B)** A graph displaying the number of embryos with GFP expression in the branchial arch and pectoral fin tissues out of all live embryos at 72 hpf. **(C)** Zebrafish enhancer assay results for *eTw-8* segments: The full sequence of the p300 ChIP-seq peak drove GFP expression in the branchial arch, while segment 3 drove similar GFP expression in the branchial arch.
(TIF)

S7 Fig Generating the *eTw5-7* deletion mouse model. **(A)** Top: The 23 kb deleted region that contains the *eTw-5*, *6*, and *7* enhancers and 4 exons of Hdac9. Bottom: Sequence of the deleted region. **(B)** Genotype analysis after the 23 kb deletion showed the PCR product sizes in WT (387 bp) and deletion (526 bp) mice.
(TIF)
