# Supplementary figures and images for: Unraveling the transcriptional regulation of TWIST1 in limb development
Source: PLoS Genet. 2018 Oct 29;14(10):e1007738. doi: 10.1371/journal.pgen.1007738 (PMC6233932; doi:10.1371/journal.pgen.1007738)

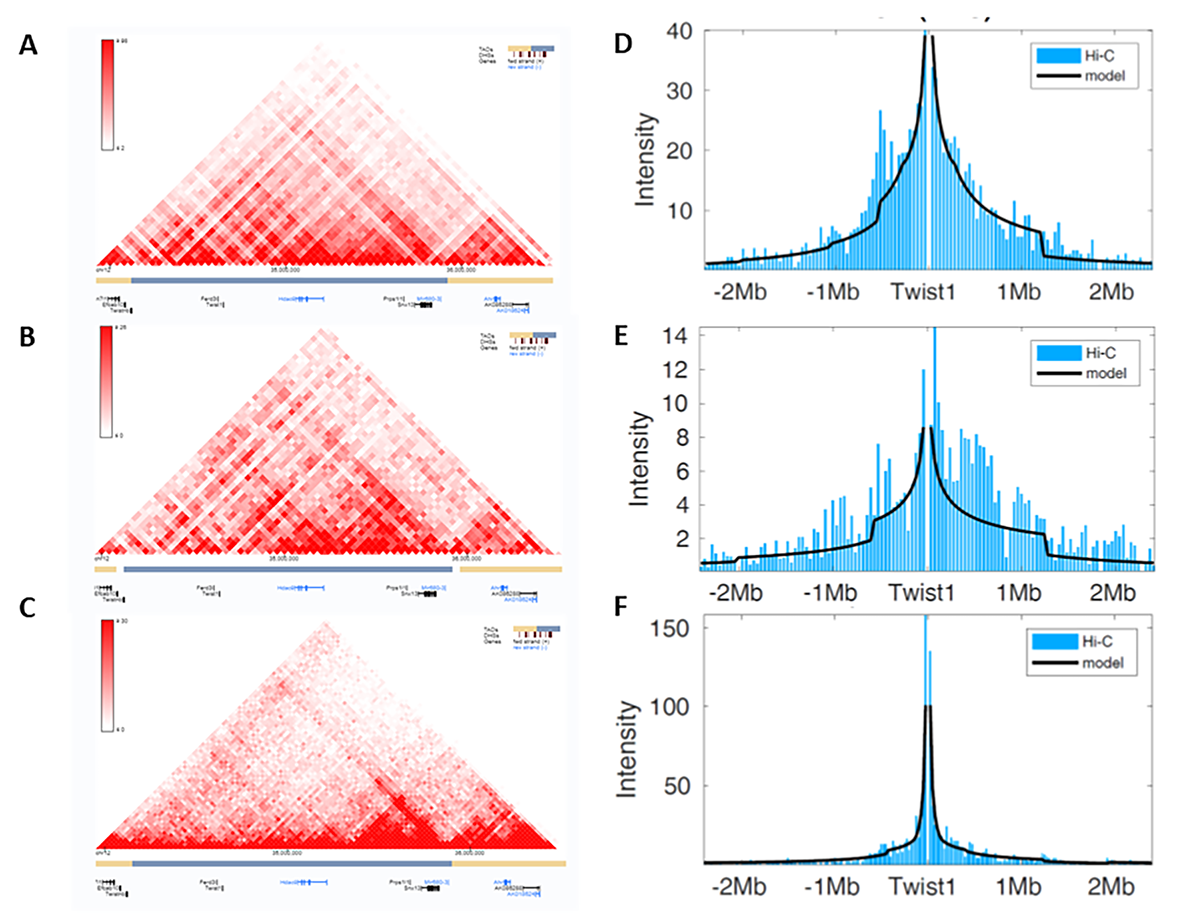

Supplement: S1 Fig — (A-C) Heat map representing the chromatin interaction frequencies from Hi-C data at the Twist1-Hdac9 locus in: (A) mouse embryonic stem cells (mESC) [22], (B) mouse cortex [22], and (C) mouse erythroleukemia CH12-LX [23]. TAD boundaries analysis by PSYCHIC for (D) mouse embryonic stem cells (mESC) [22], (E) mouse cortex [22], and (F) mouse erythroleukemia CH12-LX [23]. The Twist1 and Hdac9 genes reside in the same TAD across the different cell types. Each column represents the interaction intensity in a 50 kb window. (TIF) [file pgen.1007738.s004.tif]

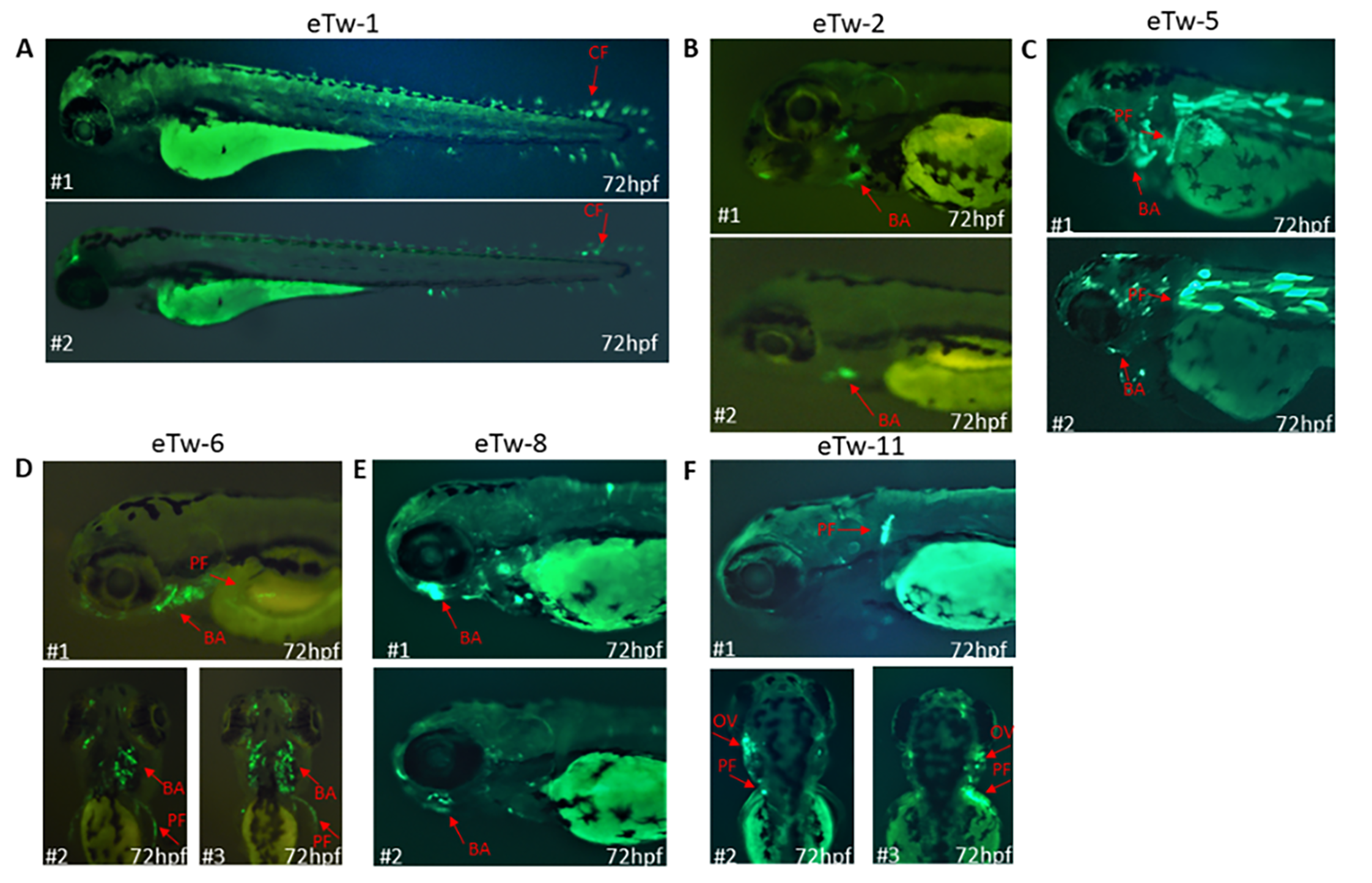

Supplement: S2 Fig — (A) eTw-1 drives GFP expression in the caudal fin. (B) eTw-2 drives GFP expression in the branchial arch. (C) eTw-5 drives GFP expression in the pectoral fin, branchial arch and somitic muscles. (D) eTw-6 drives GFP in the pectoral fin and branchial arch. Top: lateral view. Bottom: ventral view. (E) eTw-8 drives GFP in expression in the branchial arch. (F) eTw-11 drives GFP expression in the base of the pectoral fin, in the branchial arch and in the otic vesicle. Top: lateral view. Bottom: dorsal view. (TIF) [file pgen.1007738.s005.tif]

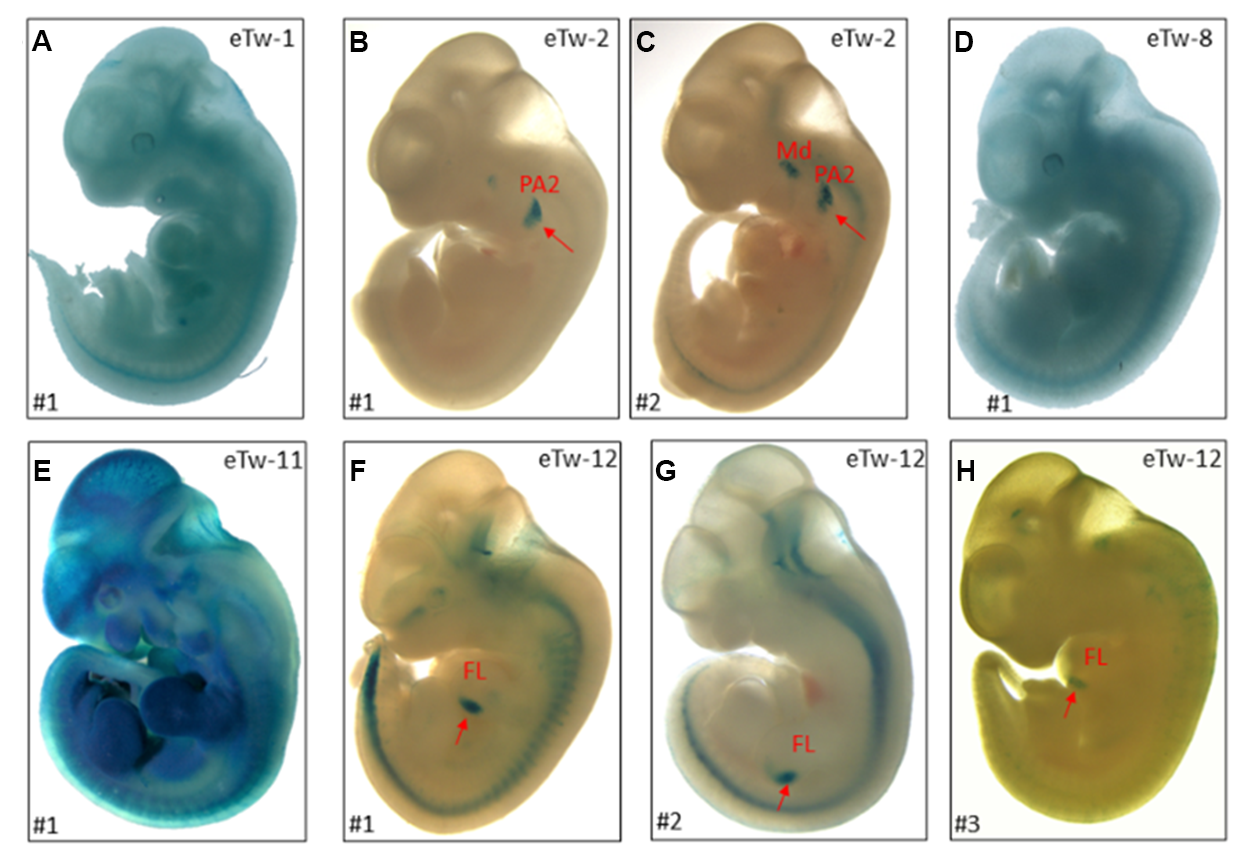

Supplement: S3 Fig — (A) eTw-1 does not show LacZ expression. (B, C) eTw-2 drives LacZ expression in the branchial arch. (D) eTw-8 does not show LacZ expression. (E) eTw-11 drives LacZ expression in the branchial arch and limb buds. (F-H) eTw-12 drives LacZ expression in the anterior limb bud. Pharyngeal arch 2 (PA2), Mandibula (Md), Forelimb (FL). (TIF) [file pgen.1007738.s006.tif]

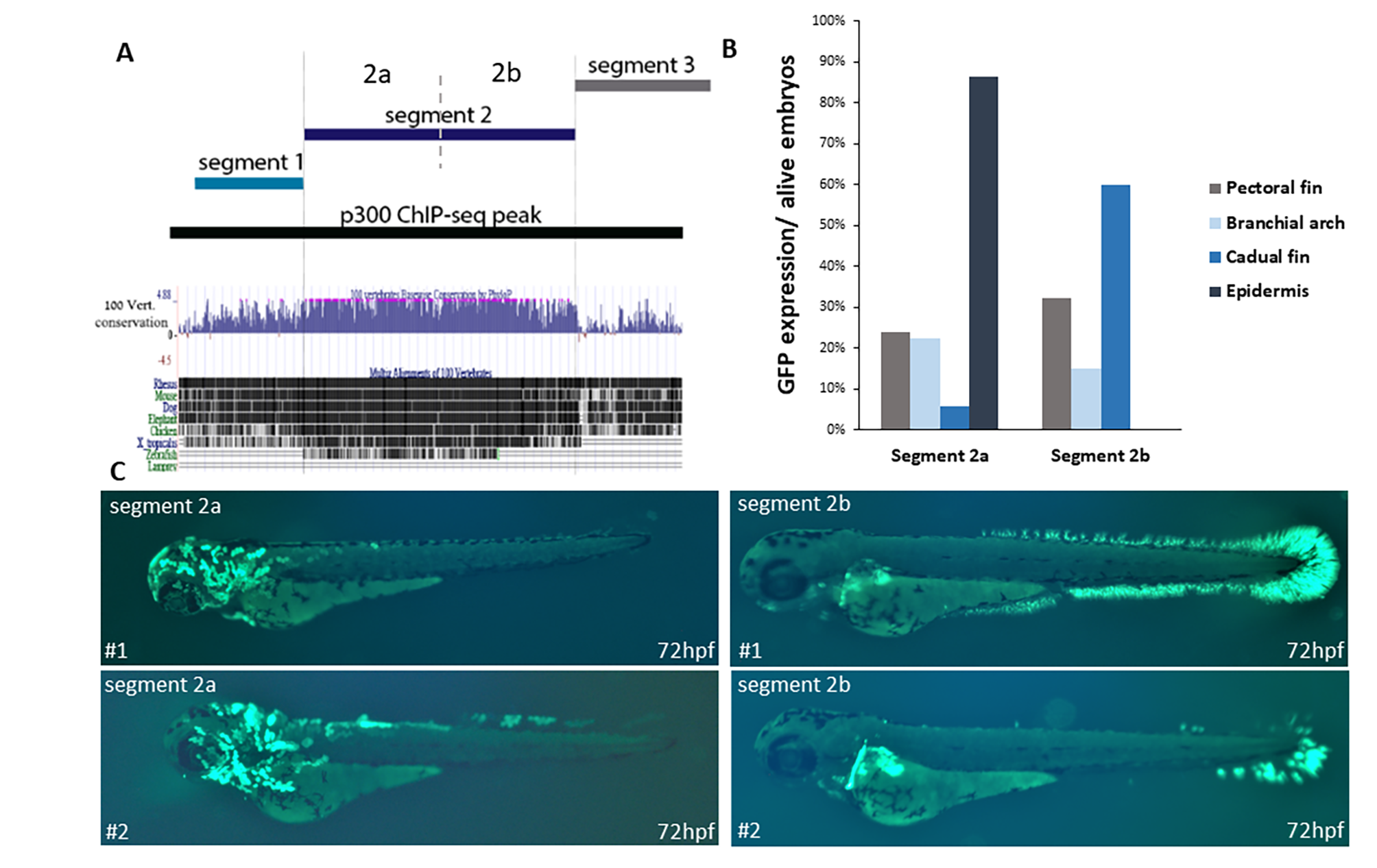

Supplement: S4 Fig — (A) The eTw-5 segment 2 was divided into segments 2a and 2b. (B) A graph displaying the number of embryos presenting GFP expression in the pectoral and caudal fins and branchial arch out of all live embryos at 72 hpf. (C) Zebrafish enhancer assay results for eTw-5 segments: segment 2a drives GFP expression in the epidermis surrounding the head and segment 2b drives GFP expression in the pectoral and caudal fins. (TIF) [file pgen.1007738.s007.tif]

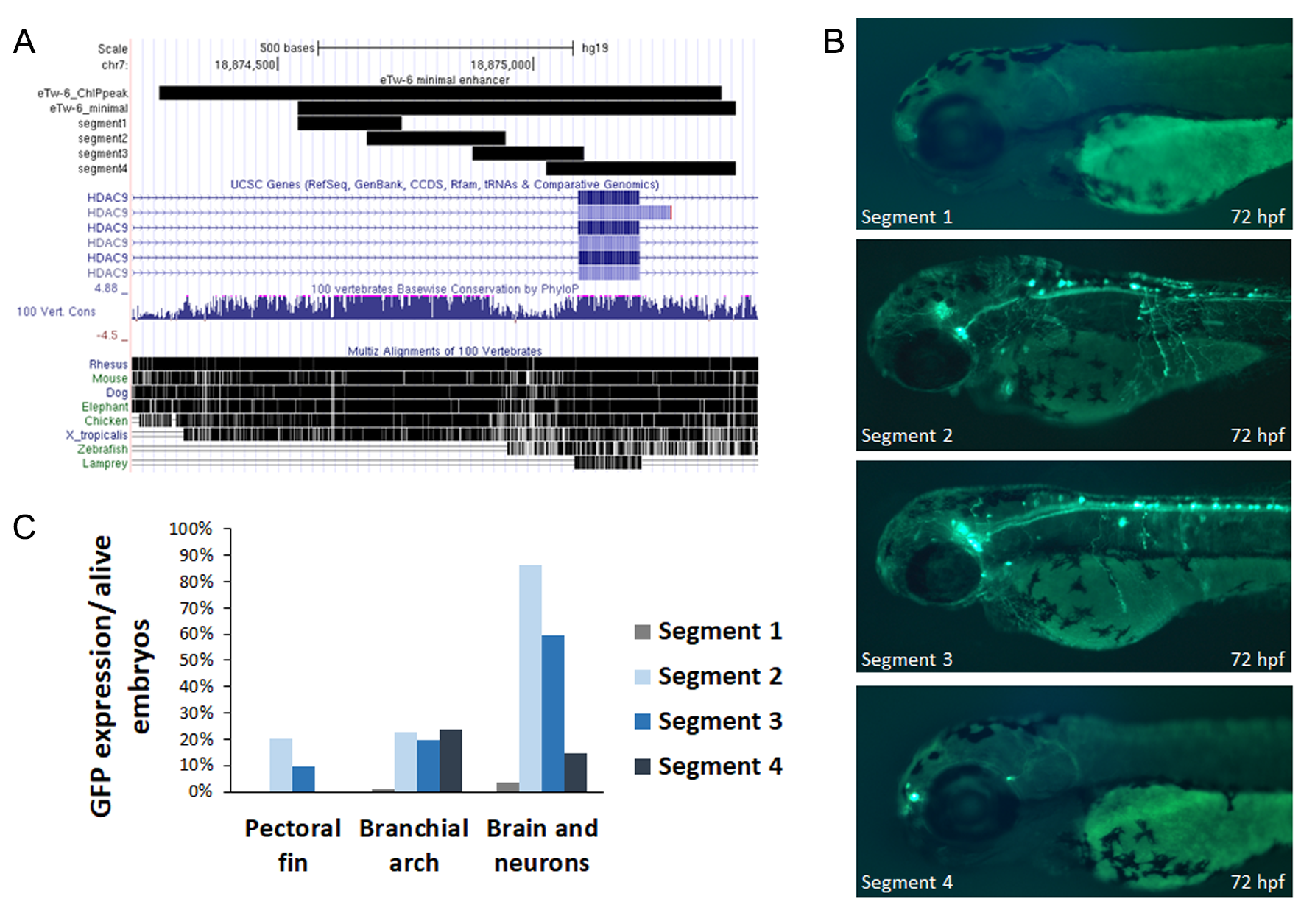

Supplement: S5 Fig — (A) The eTw-6 enhancer that drives GFP expression in the pectoral and branchial arch was divided into four overlapping segments (1–4). The UCSC genome browser (http://genome.ucsc.edu) conservation track shows that segment 4 which contains Hdac9 exon 18 is conserved between humans and fish. (B) A graph displaying the number of embryos with GFP expression in the pectoral fin, branchial arch, brain and specific neurons out of all live embryos at 72 hpf. (C) Zebrafish enhancer assay results for eTw-6 segments: segment 1 and segment 4 did not drive GFP expression, while segments 2 and segment 3 drove GFP expression in neurons near the eyes that project to the trunk. (TIF) [file pgen.1007738.s008.tif]

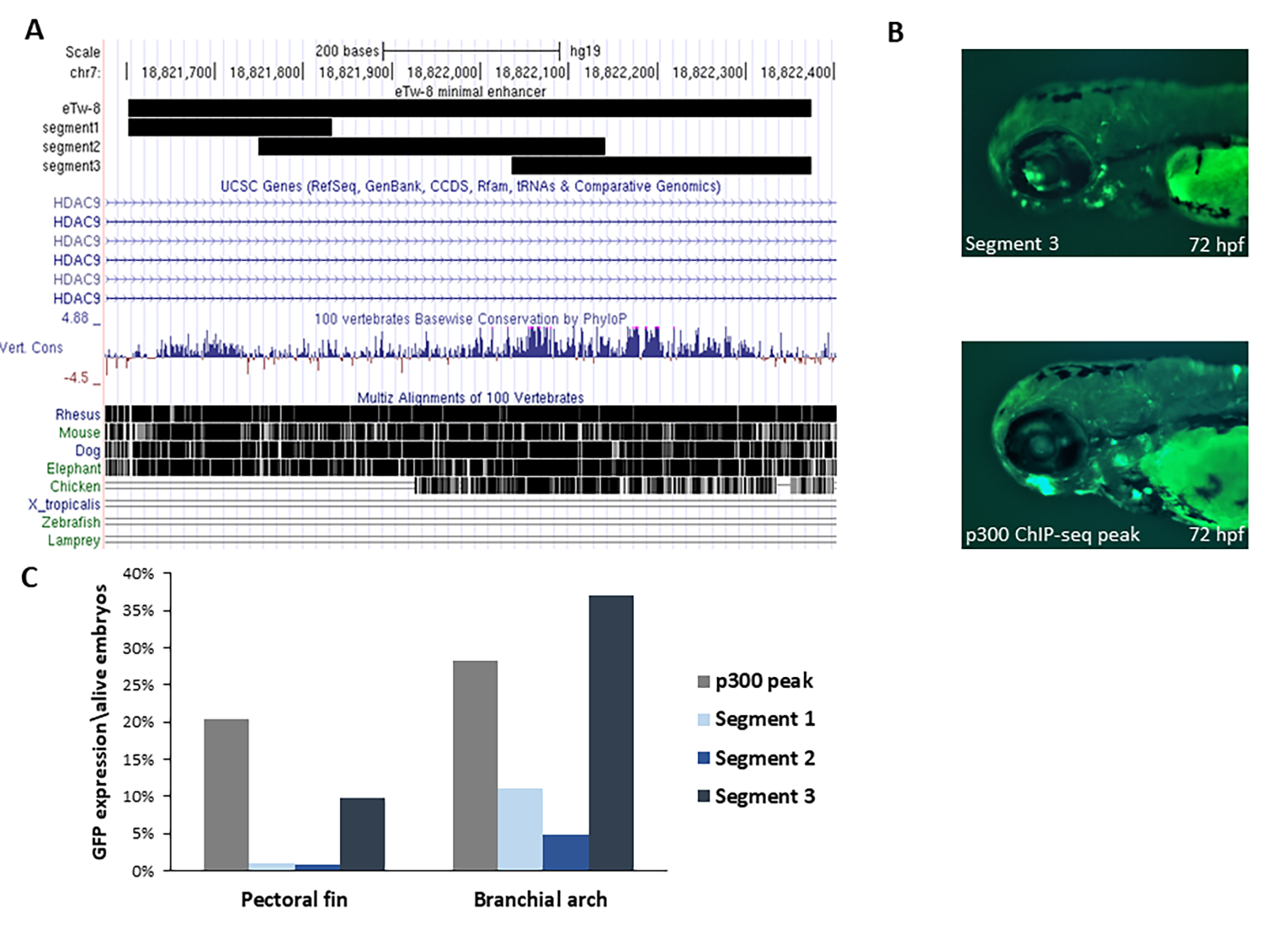

Supplement: S6 Fig — (A) eTw-8 was divided into three segments (1–3). The UCSC genome browser (http://genome.ucsc.edu) conservation track shows that segment 3 is the most evolutionarily conserved. (B) A graph displaying the number of embryos with GFP expression in the branchial arch and pectoral fin tissues out of all live embryos at 72 hpf. (C) Zebrafish enhancer assay results for eTw-8 segments: The full sequence of the p300 ChIP-seq peak drove GFP expression in the branchial arch, while segment 3 drove similar GFP expression in the branchial arch. (TIF) [file pgen.1007738.s009.tif]

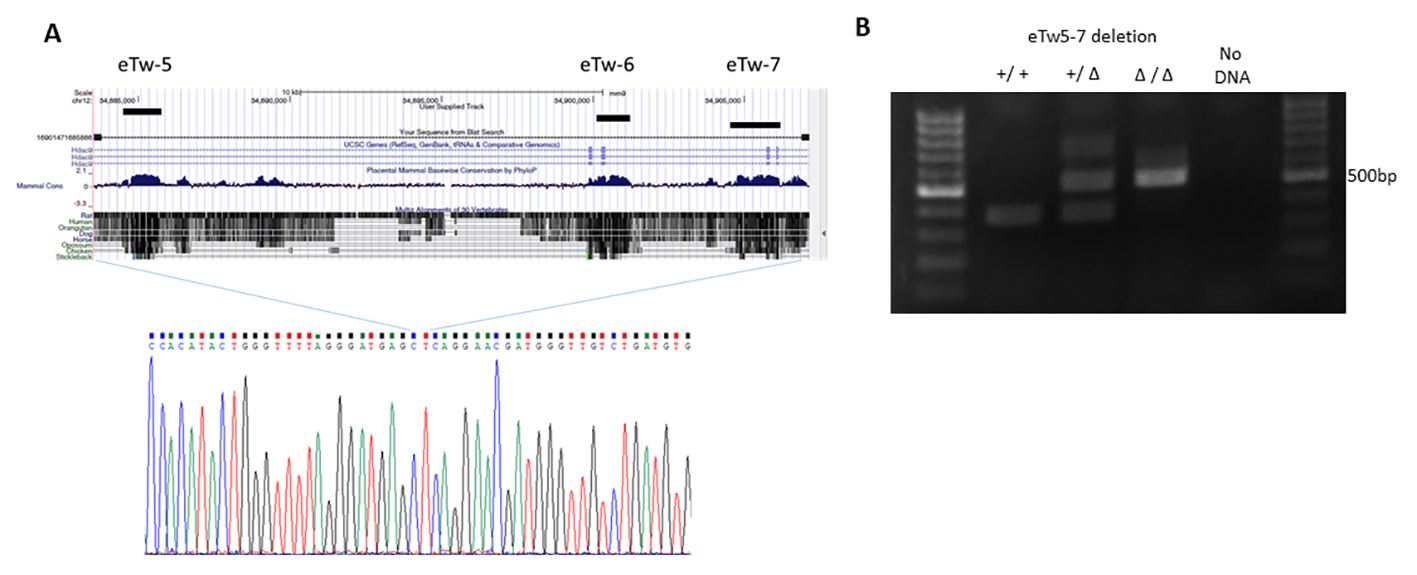

Supplement: S7 Fig — (A) Top: The 23 kb deleted region that contains the eTw-5, 6, and 7 enhancers and 4 exons of Hdac9. Bottom: Sequence of the deleted region. (B) Genotype analysis after the 23 kb deletion showed the PCR product sizes in WT (387 bp) and deletion (526 bp) mice. (TIF) [file pgen.1007738.s010.tif]
